# Curcumin Nanofiber PCL/PLGA/Collagen Enhanced the Therapeutic Efficacy of Mesenchymal Stem Cells against Liver Fibrosis in Animal Model and Prevented its Recurrence

**DOI:** 10.7150/ntno.81019

**Published:** 2023-03-13

**Authors:** Gehan Abd-Elfatah Tawfeek, Hend Ahmed Kasem, Sherif Elsayed Elshoala

**Affiliations:** 1 Clinical Pathology Department, Faculty of Medicine, Menoufia University, Egypt; 2 Pathology Department, Faculty of Medicine, Menoufia University, Egypt; 3 Internal Medicine, King Abd-Elaziz Hospital, Saudia Arabia

**Keywords:** MSCs, Curcumin/nanoscaffold, hepatic fibrosis model, ameliorative effect, preventive effect

## Abstract

The aim of this study is preconditioning of hBM-MSCs using curcumin modified nanomembrane to optimize therapy of hepatic fibrosis and preventing its recurrence.

**Methods:** The nanomembrane was prepared by electrospinning technique and characterized using conventional method (cur^-^ nanoscaffold and cur^+^ nanoscaffold). Kinetic release of curcumin was also measured by spectrophotometry. MSCs were isolated from human bone marrow (hBM-MSCs) and cultured on the both nanoscaffolds.

We evaluated the *in-vivo* effect of hBM-MSCs from both nanoscaffold cultures (cur^-^ nanoscaffold/hMSCs and cur^+^ nanoscaffold/MSCs) on liver fibrosis from its effective and preventive points and we assessed the mechanisms of these effects as *in vitro* studies as cell proliferation, its effect on hepatogenic differentiation, its effect on paracrine release of hBM-MSCs and *in-vivo* studying the effect on cell migration, survival, engraftment, fate of transplanted cells, modifying the fibrogenic and inflammatory microenvironments.

**Results:** The results of animal model showed that single injection of preconditioning of hBM-MSCs using curcumin modified nanoscaffold ameliorate the fibrosis and prevent its recurrence until 24 weeks of therapy in contrast to improvement but not ameliorative effect of hBM-MSCs/ curcumin negative nanoscaffold which recurred progressively after 12 weeks of therapy. These effects of curcumin modified nanoscaffold were results from its highly efficacy on cell proliferation, *in-vitro* and *in-vivo* hepatogenic differentiation, increasing cell migration, engraftment and survival in the inflammatory microenvironment which was markedly improved by down regulation of inflammatory mediators and upregulation of anti-oxidant factors.

**Conclusion:** hBM-MSCs cultured on the prepared curcumin nanomembrane in this study is promising in regenerative therapy for ameliorating the hepatic fibrosis and to prevent its recurrence.

## Introduction

The liver plays a pivotal role in detoxification, immune response, metabolism, and homeostasis. Many causes such as hepatitis, alcoholism, and cholestatic disease can cause liver cell damage leading to hepatic fibrosis, eventually leading to liver cirrhosis and liver failure 1.

End stage liver disease is a leading cause of death all over the world and liver transplantation is considered the best choice but it has some limitations as long waiting list, long periods of immune suppression and the poor outcome in patients not supported by liver transplantation, all these limitations give the way for cell transplantation as an alternative approach for treatment of hepatic cirrhosis 1.

Egypt has the highest prevalence of hepatitis C virus in the world, up to 20 % in some areas 2. So, Egyptian researchers have to conduct research on human stem cells to differentiate into hepatocytes. Over the past years Mesenchymal stem cells (MSCs) emerged to regenerated the damaged hepatic cells as they have the advantages of self-renewal, the ability to differentiate into specific cell types and secretion of various growth factors as hepatocyte growth factor (HGF), Vascular endothelial growth factor (VEGF). These properties make them very promising in tissue regeneration and repair (3-4). However, decreasing their survival and engraftment duration by the inflammatory environment and oxidative stress in liver fibrosis limits their therapeutic efficacy 5. So, preconditioning to provide cytoprotective stimuli in the MSCs to promote their survival in the inflammatory microenvironment supposed to be advancement in regenerative medicine. Recently, many approaches have been introduced, such as genetic modification and pre-incubation with pharmacological/- chemical agents, trophic factors/cytokines and a hypoxic condition, in order to further enhance the therapeutic potential of transplanted MSCs 6-9. These studies proved that primed MSCs have more therapeutic effects than wild MSCs. Curcumin (Cur), also known as [1,7‐bis(4‐hydroxy‐3‐methoxyphenyl)‐1,6‐heptadiene‐3,5‐dione] or diferuloylmethane, is one of the main natural compounds present in the rhizome of Curcuma longa and other Curcuma spp. 10. Several studies investigated the therapeutic effect of the Cur and its various formulations due to its pharmacological properties as antioxidant, anti‐inflammatory, anticoagulant, anti- atherosclerotic, antibacterial, antifungal, antiviral and anti-oncogenic effects 11-13.

Previous studies reported the ability of Cur to improve healing wounds 14, bone regeneration 15, and inflammatory diseases as rheumatoid arthritis and osteoarthritis 16 and cancer 12. The main limitations of curcumin are low hydrophilicity and poor bioavailability 17. Multiple formulations as hydrogels, fibers, emulsions and nanostructures are supposed to overcome these limitations 17. Recently, nanobiotechnology as electrospinning provides promising approaches for delivery of drugs with poor bioavailability, nanofibers using both natural and synthetic combinations with different properties in recent years are highly promising for drug delivery 18.

The current study aimed to provide combined nanofibers construct to deliver the cur supposing that the control release of cur will improve its bioavailability so, preconditioning of human bone marrow MSCs (hBM-MSCs) with this cur- nanofiber will increase its efficacy to ameliorate animal model of liver fibrosis.

## Materials and Methods

This research was done in Clinical Pathology and Pathology Departments, Faculty of Medicine- Menoufia University, from March 2021 to June 2022.

### Preparation of curcumin nanoscaffold

Two solutions were prepared, first poly lactic-co-glycolic acid (PLGA, 85:15, molecular weight = 50,000; Sigma) was dissolved in acetone and water, stirred for 6 hours at room temperature and Polycaprolactone (PCL, molecular weight = 65,000; Sigma-Aldrich, St. Louis, MO, USA) was dissolved in a mixture of dimethylformamide (DMF) and chloroform (1:9, vol:vol) and stirred for 6 hours at room temperature. PCL solution added to the PLGA solution (1:1) under magnetic steering of 100 rpm at 25 °C (solution 1). Solution 1 stirred for overnight at room temperature to obtain uniform solution. Solution 2 (curcumin/collagen): 1 g collagen with or without 5 mg curcumin were dissolved in 2,2-Trifluoroethanol (8% w/v) and stirred for overnight at room temperature.

The two scaffolds (cur^-^nanoscaffold & cur^+^nanoscaffold) then were fabricated using electrospinning technology: the polymers solution was placed in two 5mL plastic syringes fitted with a needle with a suitable tip diameter, nanofiber with/without curcumin were fabricated by solutions 1&2 placed simultaneously at the electrospinning instrument. The applied voltage for the collagen/curcumin mixture was 20 and 15 kV for PCL/PLGA mixture with flow rate was 0.2 mL/h and the syringes tip at distance 15 cm from the collector 17.

### Characterization of curcumin nanoscaffold

After drying with absolute ethanol and sputter coating with 5 nm gold, the diameter and the distribution of the electrospuned PLGA nanofiber scaffold were measured, examined by Scanning electron microscopy (SEM) (JEOL JSM-6510LV) before and after seeding of the cells.

The average diameter of nanofibers was determined by using Image J analysis software. Measurements of the zeta potential were done using a zeta-sizer (Malvern, UK) at 25 °C in order to evaluate the surface charge of the nanomaterials. The hydrophilicity was detected by water absorption test, for this: 0.5cm squared parts of the membrane were cut and the dry weight was measured (W0) then the samples (3 samples for each time point) were incubated in 6-well plate filled with phosphate buffered saline (PBS; Sigma‐Aldrich, St. Louis, MO) (2 mL/sample) for 72 hours at 37°C while shaken at 60 rpm. After that the samples were weighed after 24, 48, and 72 hours and the final weight was considered as the wet weight (Wn). The water uptake (%) was calculated as follows:

Water uptake (%) = [(w_n_-w0) /w0]x100

#### *In vitro* Cur release

2cm of the scaffolds were put in 10ml P.B.S (pH 7.4 and 37°C), 2ml of the media was removed at 1hr, 2hr, 4hrs, 12hrs;1, 2, 4,8, 12day time points and each time was replaced by the same amount of fresh medium, cur was measured in the removed medium using spectrophotometer (Beckman Coulter) at wavelength of 450 nm.

### Isolation and characterization of human BM derived MSCs (hBM-MSCs)

This study included patients with normal bone marrow, patients with hypoplastic or bone marrow infiltration were excluded. Under complete aseptic conditions the bone marrow was diluted with sterile PBS in the ratio of 1:1. The diluted blood was layered on ficoll-hypaque at ratio one volume of ficoll: two volumes of blood then, the tubes were centrifuged for 20 minutes at 1800 rpm. The mononuclear cells (MNCs) fraction was collected, washed twice, count and viability was done using trypan blue (Sigma). The MNCs suspensions were plated at a concentration of (1×10^6^ cells/ml) and allowed to adhere to tissue culture plastic flasks 25cm^2^, incubated at 37 C 5% CO2 in fresh complete nutrients medium which includes: DMEM-LG with L-glutamine, 10% F.B.S, Penicillin-strptomycin (10,000 U/ml and 10,000 µg/ml), Fungizone (250 µg/ml), all from Lonza. Then the media was changed twice weekly until reaching 70% to 90% confluence. After that, the adherent cells were harvested by trypsin-EDTA 0.25% solution 30. Sub-cultured passage three was used for identification and dither cells were identified by microscopic examination and flowcytomeric analysis, for this 1x10^6^ cells were incubated in 2% FBS/PBS at 4°C for 30 min with 10 μl of monoclonal antibody specific for CD45, CD34, Oct3/4, CD44 and CD90 (BD Pharminogen).

### Cell proliferation and biocompatibility test

hBM-MSCs cultured on scaffolds in 96 well plates with the complete culture medium and incubated the membranes at 37 °C in a 5% CO2 for 12 days. Cell proliferation was assessed by measurement of light absorbance at 450 using Cell counting kit, cck-8 (Sigma-aldrich).

### Animal model and treatment

138 Albino female rats of local strain with average weight of 225-300 gm were used in this study. Rats were purchased from the military animals farm (Cairo). Rats were housed in a fully ventilated cages with free access to water and balanced diet 12-hour (h) light/dark cycles. The animal husbandry and welfare aspects were guided by National Institutes of Health for use of laboratory animals. The experiment was approved from review board committee (IRB: 1/2023PATH20-2), faculty of medicine, menoufia university which in compliance with the National Institutes of Health for use of laboratory animals.

**Study design**: Chronic Liver fibrosis was induced in rats by intra-peritoneal injection of CCl_4_ in a dose of 1 ml/kg diluted by olive oil 1:1 twice a week for 8 weeks 21. Then, maintenance dose once weekly was given up to 24^th^ week of experiment.

To prove the incidence of fibrosis, 5 animals were anesthetized with chloroform before scarification 24 hours after the administration CCl_4_ at 4^th^ week, and at 8^th^ week and the liver of each was excised for histopathology to confirm the occurrence of fibrosis.

The remaining experimental Animals were divided into the following four groups (the study is completely randomized study): Group I" Normal control (32 rats): The rats received the same volume of olive oil alone. Group II: CCl_4_ group" (32 rats): rats were injected with CCl_4_ plus single intravenous injection of 0.9% saline in the rat tail vein. Group III (32 rats): " CCl_4_ plus wild hBM-MSCs (cur^-^ nanoscaffold/MSCs treated group) in a dose of 10 x 10^6^ P3 cells/rat 30. Group IV (32 rats): " CCl_4_ plus preconditioned hBM-MSCs (cur^+^ nano scaffold/MSCs treated group) in a dose of 10x 10^6^ P3 cells/rat 30. The cells were injected 24hrs after the end of the 4^th^ week of CCl_4_ injection. The animals were investigated in 4 different time points (8 rats at each time point) 1^st^ week, 4^th^ week, on 12^th^ week and 24^th^ week of cell injection.

Blood samples were collected from the retro-orbital venous plexus, using a fine heparinized capillary tube introduced into the medial epicanthus of the rat's eye 22. The supernatant serum was collected for biochemical studies.

- The livers were excised, for each sample one lobe of the liver was fixed in 4% formalin and routinely processed ending with paraffin embedding and prepared for histopathological and immunohistochemical studies. The other lobe was preserved in RNA Later at - 80 °C for PCR.

### Evaluation of stem cell therapy with and without curcumin nanoscaffold in chronic liver fibrosis model

#### Biochemical analysis

Blood samples were collected from the retro-orbital venous plexus using a fine heparinized capillary tube introduced into the medial epicanthus of the rat's eye. The supernatant serum was collected in dry clean tubes and was used for estimation of serum ALT (Alanine transferase), AST (Aspartate transferase) albumin and total bilirubin measurement (Cobasintegra 400 plus, No. 500281).

#### Histopathological examination

Hematoxylin& Eosin (H&E) stained sections were analysed for evaluation of degree of fibrosis under optic microscopy. Masson trichrome (MT) staining for confirmation of the H& E stain results 36.

#### Quantitative analysis of liver fibrosis

- By MT staining: Liver sections cut at 10 µm were fixed with 4% paraformaldehyde in a 0.1 M phosphate buffer for 20 minutes and then washed with a 0.1 M phosphate buffer. Tissue sections were stained with 0.1% MT, and this was followed by dehydration; a coverslip was applied with Permount. We quantified the area of liver fibrosis with MT staining, using an Olympus microscope. The stained area, was analysed by computer-assisted image analysis with Image-Pro software. The mean of the fibrotic area was obtained from 7 randomly selected regions per section from a total of 7 sections in each rat and was expressed as a percentage 36.

- By hydroxyproline content: 10 mg tissue was homogenized in 100 µL of water was transferred to 2.0 mL polypropylene tube. 100 µL of concentrated hydrochloric acid was added, cap tightly, and hydrolysed and homogenized at 120 °C for 3 hours. 50 µL of supernatant then transferred to a 96 well plate. hydroxyproline concentration is determined by the reaction of oxidized hydroxyproline with 4-(Dimethylamino) benzaldehyde (DMAB), which results in a colorimetric (560 nm) product, proportional to the hydroxyproline present which is expressed by µg / µL 37.

- On molecular level by collagen type 1 mRNA level by qRT-PCR as described below.

### Effect of cur^+^ nanoscaffold preconditioning of hBM-MSCs on *in-vitro* hepatogenic differentiation

The hBM-MSCs were seeded on sterile nanoscaffolds (cur^+^ nanoscaffold and curc^-^ nanoscaffold) in 35mm petri dishes for 24 hours allowing the cells to be adherent to the membrane then the media was exchanged with the differentiation media for 14 days constituted of DMEM L/G 10% FBS, penicillin/streptomycin, HGF 20ng/ml and b-FGF 10ng/ml (R&D systems) 38. The hepatocyte -like cells were identified by morphology, hepatic gene expression of AFP, ALB, CK18, HNF4α, A1AT, CYP1A1 and HGF by qRT-PCR and measurement of AFP, albumin, transferrin, urea and fibronectin (AFP measured using a chemiluminescence immunoassay kit; for ALB production using immunoturbidimetric method by microalbumin kit and transferrin was done using immunoturbidimetric method by cobas 6000 - module c501 analyzers); for urea production: after treatment of differentiated cells by NH4Cl (5 mM) in 1 ml of fresh medium for 24 h. urea production was measured using the Urea Assay in the supernatant using a colorimetric assay kit; fibronectin by ELISA (Biovision). All the measurements were done at day7 and 14 of differentiation. Cells used for the assays were trypsinized and counted and the results were normalized based on 10^6^ live cells.

### Analysis of cell migration and engraftment

*In vitro* wound healing test: 10,000 hBM-MSCs were seeded on scaffolds placed in 24 well -plate and when reached 90% confluence, scratch wound was done with a micropipette and filling of the wound was followed every 4 hours under inverted microscopy. Also, the cells migrated to the wound were counted/ HPF 34.

#### *In-vivo* by detection of chimerism in female rats by qRT-PCR

DNA was extracted from liver cells using Thermo Scientific GeneJET Genomic DNA Purification Kit (Thermo Fisher Scientific, MA, USA) according to the attached instructions.

PCR assay was performed as previously described 13 using the specific primers and probes for human sex-determining region Y (SRY) gene (Thermo scientific). The PCR reaction was in a total volume of 25 μL containing 12.5 μL of taqman universal master mix (Applied Biosystem), PCR cycling conditions were 5 min at 95 °C, followed by 40cycles of 15 s at 95 °C, 1min at 59 °C for annealing, β-actin was used as positive control of DNA quality Primers and probe are showed in table 1. Quantitation of the male DNA in the recipient female rats by construction of standard curve which was done by serial eight dilutions from 1:10 to 1: 100,000 of starting concentration of DNA extracted from male mononuclear cells and measured by spectrophotometer, each dilution was done in triplicate. Human male DNA was used as positive control, negative control was done from PCR mix without DNA and from female DNA. The test done at 1,4, 12 and 24 week of cell infusion.

### Detection of distribution, survival and fate of transplanted cells

To evaluate the distribution and survival of transplanted cells, human-specific proliferating cell nuclear antigen (PCNA) (Novus biological) was assessed by immunohistochemical staining. Enough dual enzyme block was applied to cover the specimen, incubated 5-10 minutes. excess buffer was tapped off and then diluted primary antibody (1:100) to cover specimen, at 4°C for 18 hours, washed again with 0.1 M PBS for 5 minutes 3 times, reacted with fluorescein-conjugated goat anti-mouse immunoglobulin G (1:50) at room temperature for 1 hour and washed with 0.1 M PBS for 5 minutes 3 times. chromogen solution was applied to cover the specimen, incubated for 5-30 minutes with ACE+ or 5-10 minutes with DAB+, rinse gently with distilled water. The slides were immersed in hematoxylin counter stain, Sections were then washed and mounted with anti-fading medium before con-focal microscopic examination 36.

To detect the fate of transplanted cells if differentiated into functioning hepatocytes, human specific AFP and Hep Par-1 by immunostaining (Human kit, Germany), in addition to gene expression of human specific of AFP, CK-18 and ALB.

### RNA extraction and qRT-PCR

The effect of curcumin nanoscaffold on regeneration of the liver at molecular level was evaluated via estimation of key genes of liver fibrosis (α-SMA MMP-9, TGFβ1, FGF2), hepatogenic differentiation genes, inflammatory genes and Superoxide dismutase by qRT-PCR: total RNA was isolated from cultured hMSCs and from the liver tissues of treated groups using RNeasy Plus Mini Kit (Qiagen) according to the manufacturer's instructions. Real-time reverse transcriptase polymerase chain reaction (qRT-PCR) was performed as previously described **(**9). Two-Step PCR was done, 8μL of RNA was reverse transcribed into cDNA using MultiScribe Reverse transcriptase according to manufacturer's protocol using GeneAmp Gold RNA PCR Reagent Kit (Applied Biosystem). Volume of 5 μL of cDNA was added to a final PCR reaction mixture of 25 μL containing 12.5 μL Master Mix SYBR Green Dye (Applied Biosystem), 1.5 μL of each Primer, 4.5μLRNase free water. Cycling conditions 2 min at 94°C as a first denaturation step, followed by 40 cycles of 15 s at 94°C, 30 s) and 30 s at 72°C.The panel primers and conditions used in this study were showed in table 1.

### ELISA for cytokine and trophic factors array

Serum level from experimental animals for IL-6, TNF-α, Il-10, SOD-1 and culture supernatant for assessment of HGF, EGF and VEGF (all from Invitrogen) were detected by enzyme-linked immunosorbent assay (ELISA). A target-specific antibody has been pre-coated in the wells of the supplied microplate. Samples, were then added into these wells and bind to the immobilized antibody. The sandwich is formed by the addition of the second antibody, a substrate solution is added that reacts with the enzyme-antibody-target complex to produce measurable signal. The intensity of this signal is directly proportional to the concentration of target present in the specimen 45.

### Statistical analysis

The collected data were analysed using SPSS software (version 20.0). Descriptive statistics in the form of the mean ± SD were used for parametric data. The differences between the variables were evaluated by the chi-square test, Kruskal-Wallis test and one-way analysis of variance according to the data. Student's t-test, ANOVA, Fisher's exact test and the Mann-Whitney test were used. The association between genotypes and disease was assessed by computing the odds ratio (OR) and 95% confidence interval. Regression analysis was used to estimate the relationships among variables. The significance level was set at 0.05 or less.

## Results

### Characterization of cur/collagen/PCL/PLGA nanoscaffold

**Figure 1:**
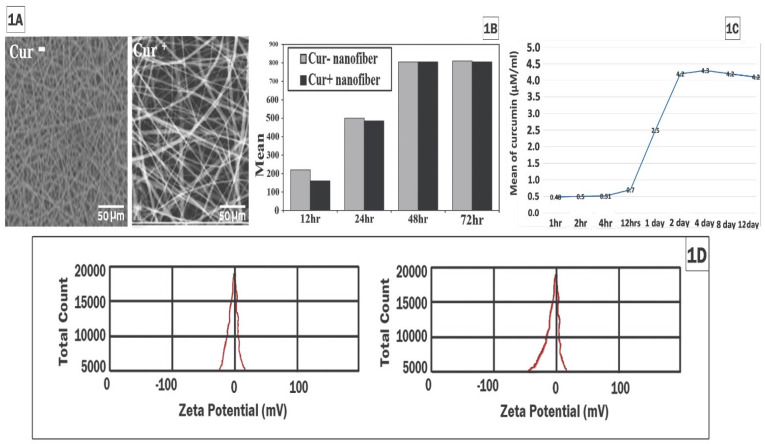
Characterization of cur/collagen/PCL/PLGA nanoscaffold: SEM examination of the scaffold (fig.1A); hydrophilicity of the scaffold (fig.1B); curcumin release kinetic (fig.1C); Zeta potential of the scaffolds (fig.1D).

Electrospuned nanofiber scaffolds when examined by SEM showed randomly distributed fibers with diameters 300-320nm in cur^-^ and 600-840 nm in cur^+^ with different size of pores and high pore density (Figure 1A). At 12 hr, cur^-^ has more hydrophilicity than cur^+^ and there was no significant difference at the other time points between cur^-^ and cur^+^nanoscaffold (Figure 1B). Zeta potential showed negative surface charge for both nanofiber which the negativity was more in cur^+^ (-43.56 mV) than cur^-^ (-26.76mV) (Figure 1D).

Curcumin release kinetic from the scaffold was showed in Figure 1C, there was low concentration of curcumin during the first 24hrs then the release was increased at 24hr with progression increase until the 2nd day and this increase may be due to increase the hydrophilicity over the time, after that become constant until the 12 days period of culture.

### Characterization of hBM-MSCs

**Figure 2:**
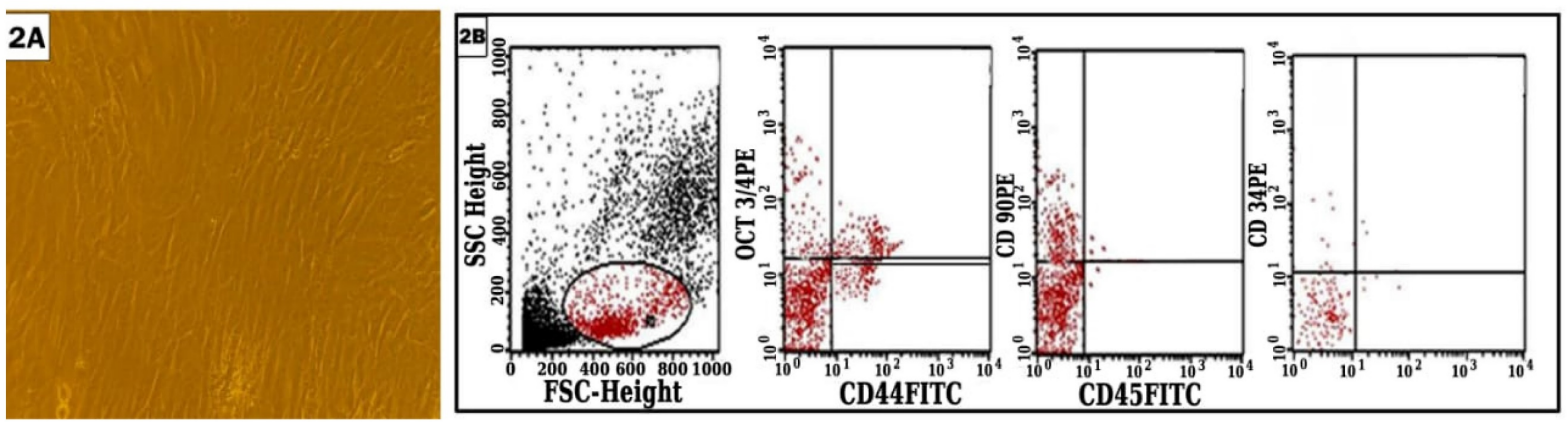
Flowcytomeric analysis of the isolated cells.

MSCs were successfully isolated from all B.M samples with viability 96-100% showing adherence to plastic surface and fibroblastoid morphology. Using flowcytometry, the cells were positive for Oct3/4, CD44, CD90 and negative or low expression to CD34 and CD45 (Figure 2).

### Ameliorative and protective effect of curcumin nanoscaffold on liver fibrosis

We compared non treated (CCL_4_) and treated groups after 1,4,12 and 24 weeks of treatment regarding biochemical analysis, detect level of fibrosis by histopathological analysis, molecular and hydroxyproline content).

#### Biochemical analysis

**Figure 3:**
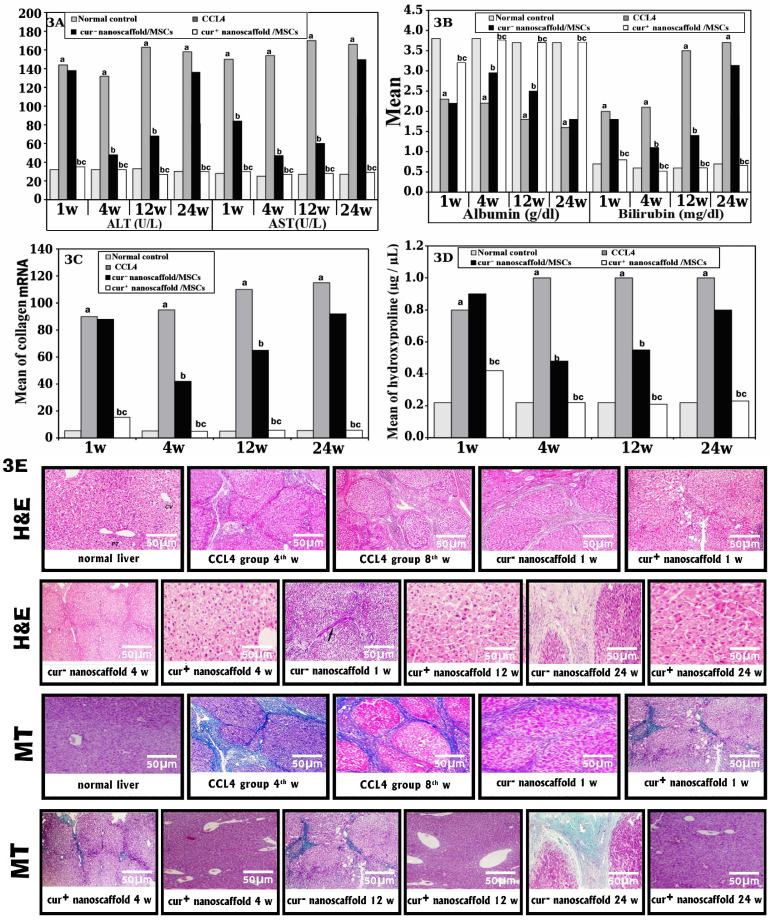
Effect of curcumin nanoscaffold on liver fibrosis: Biochemical analysis of studied groups (fig.3A&B); mRNA of collagen (fig.3C); hydroxyproline assay (fig.3D); histopathological examination by H&E and MT (fig.3E).

Serum levels of ALT, AST and total bilirubin were significantly increased with decreased of ALB in CCL_4_ group compared with normal control (p < 0.001) and there was significant difference between non- treated and treated groups (p < 0.001). Also, cur^+^nanoscaffold/MSCs showed significantly lower levels of liver enzymes and bilirubin and higher level of albumin that return to normal values after one week of treatment and this result was remained until the end of 24 week of treatment compared to cur^-^nanoscaffold/ MSCs treated group (p < 0.001) which showed delayed improvement until the 4^th^ week of treatment and deteriorated again on 12 and 24 week of therapy (Figure 3 A-B).

#### Histological Examination by H&E and masson trichrome morphometric analysis of liver sections (Figure 3E)

Rat liver treated with CCl_4_ for 4 weeks showed mild to moderate fibrosis with expansion of portal tract by fibrous tissue which progressively increased at 8^th^week to massive disrupted lobular liver tissue architecture, extensive fibrosis in the form of bridging fibrosis and nodular formation at 8^th^week. In cur^-^nanoscaffold/ MSCs treated group, rat's liver showed no improvement in liver architecture on 1^st^ week of cell therapy and on 4^th^ there is mild to moderate improvement in liver fibrosis compared to CCl_4_ group but this improvement could not be seen on 12 &24 week of therapy with progression of fibrosis in 12 and 24^th^ w while in cur^+^nanoscaffold/MSCs treated group showed improvement in liver architecture on 1^st^ week which reached the normal preserved lobular liver architecture without fibrosis on the 4^th^ of cell therapy, additionally this dramatic response was observed also on 12 &24 week of therapy.

Masson trichrome stained sections were used to determine the percentage of fibrotic areas. In normal control group, the collagen fibres represent about 1.4% ± 0.3% of the total liver area. in CCl_4_ group showed intense deposition of collagen fibres in the portal tract, around central veins with bridging fibrosis and the fibrotic area reached 15.0%± 1.1% of the total liver area. In cur^-^nanoscaffold/ MSCs treated group, the fibrotic tissue was less intense than CCl_4_ group and reached about 6.5% on 4^th^ week which increased again to 8 % and 10% on 12 &24 week respectively, however in cur^+^ nanoscaffold treated there was marked decrease on 1^st^ week (3.6%) with no fibrous tissue could be detected on 4^th^ week of treatment and the percentage of collagen fibres was reached as normal control (1.5%) and this effect was maintained up to the 24 week of therapy. results were statistically confirmed by values obtained from morphometric analysis.

#### Results of qRT-PCR and hydroxyproline content

On molecular level, collagen I mRNA level showed significant increase in CCl_4_ group by 16.9 fold than the normal control (p < 0.001) which significantly reduced in cur^+^nanoscaffold/MSCs groups by 6.0 fold (p < 0.001) and dropped to normal values on 4^th^ week of therapy with no change until the end period of the experiment. In contrast to cur^-^nanoscaffold/ MSCs group, there was delayed improvement until 4^th^ week of therapy by 2.3 fold (p < 0.001) which deteriorated later on the remaining period of the experiment (Figure 3C).

Figure 3D showed the collagen content in fresh liver tissue which was quantified by hydroxyproline assay, in normal control the amount was 0.22µg / µL which increased significantly in CCl_4_ group up to 0.9 ± 0. In cur^-^nanoscaffold/ MSCs group showed reduction at 4^th^ week of treatment and re-rise again on 12&24 week of therapy. In cur^+^nanoscaffold/MSCs, the collagen content began to be improved after 1 week of therapy which dropped to normal values at 4^th^ week (0.22µg /µL) and preserved its values on 12&24 week of therapy.

### Role of curcumin nanoscaffold in cell proliferation, migration, survival and paracrine factors of hBM-MSCs

**Figure 4:**
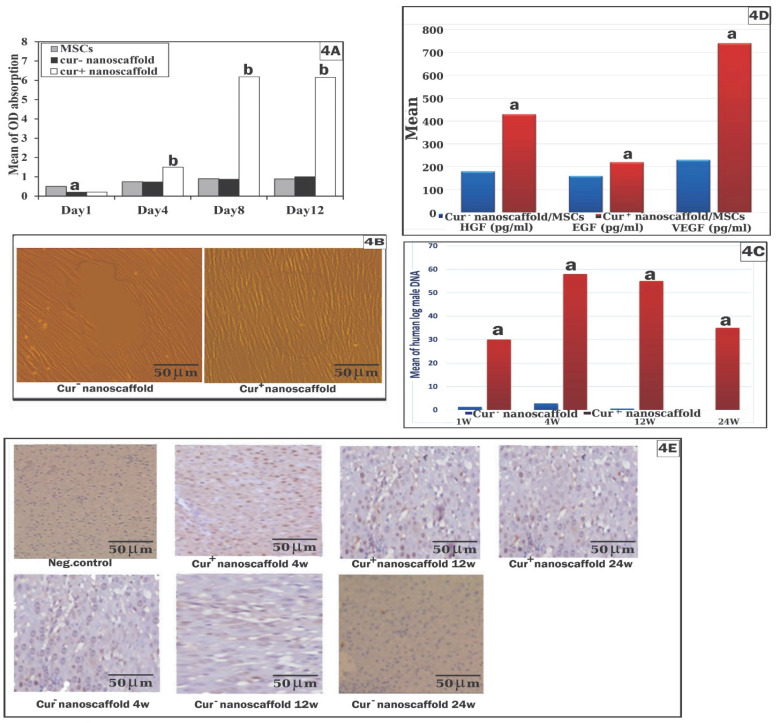
Role of curcumin nanoscaffold in cell proliferation, migration, survival and paracrine factors of MSCs: assessment of cell proliferation by cck-8 kit (fig.4A); cell migration by *in-vitro* wound test **(fig.4B)** and quantitation of human male DNA (fig.4C); paracrine factors of MSCs (fig.4D); survival of the injected cells in the liver tissue by human PCNA assay (fig.4E).

The biocompatibility and cell toxicity of the scaffold was assessed qualitatively by SEM and quantitatively by CCK-8 kit, SEM examination showed reaching the cells 90% confluence at the 2^nd^ day which became multilayer at day 4 on cur^+^nanoscaffold. However, cells reached 60%-70% at the 10^th^ day on cur^-^ nanoscaffold. By cck-8 showed increased cell proliferation after 24hrs on neutral culture plate than both nanoscaffolds. Cell proliferation on cur^-^ nanoscaffold at days 4 and 8 showed no significant difference with culture plate (p > 0.05) which increased significantly at day 12 (p < 0.05). However, cell proliferation on cur^+^ nanoscaffold showed significant increase beginning from day 4 than both culture plate and cur^-^ nanoscaffold (p < 0.001) (Figure 4A).

To assess if the preconditioning of MSCs by curcumin nanoscaffold improve its migration to the target site, the results of *in-vitro* scratch wound showed that the wound was filled completely after 12 h with presence of 300 cell/HPF in cur^+^ nanoscaffold compared to 50cells/ HPF at 12hrs and 150 cell/HPF at 24hrs in cur^-^ nanoscaffold (fig. 4B). We also evaluate the migration of cells *in-vivo* by quantification of male DNA using SRY gene in the female rats which revealed that significant higher level of male DNA in group treated with cur^+^ nanoscaffold/MSCs than cur^-^ nanoscaffold/MSCs (Figure 4C).

In cur^+^nanoscaffold/MSCs, the cells were survived up to 24 week after therapy mainly around the central vein which was observed by human specific PCNA staining and also by detection of male DNA while these findings could be detected up to 12 week in cur^-^nanoscaffold/MSCs treated group (Figure 4C&E).

To confirm if cur^+^nanoscaffold preconditioning has an impact on paracrine release of MSCs, the results of ELISA showed that cur^+^nanoscaffold preconditioning increased the capacity of paracrine release of MSCs than cur^-^nanoscaffold/MSCs as evidenced by increased release of HGF (430 ± 40 pg/ml vs 180 ±30 pg/ml, p < 0.001 ), EGF (220 ± 20 pg/ml vs 160 ± 25 pg/ml, p < 0.05) and VEGF (740 ± 50 pg/ml vs 230 ± 60 pg/ml, p < 0.001) (Figure 4D).

### Evaluation of *in-vitro* hepatogenic differentiation on curcumin nanoscaffold

**Figure 5:**
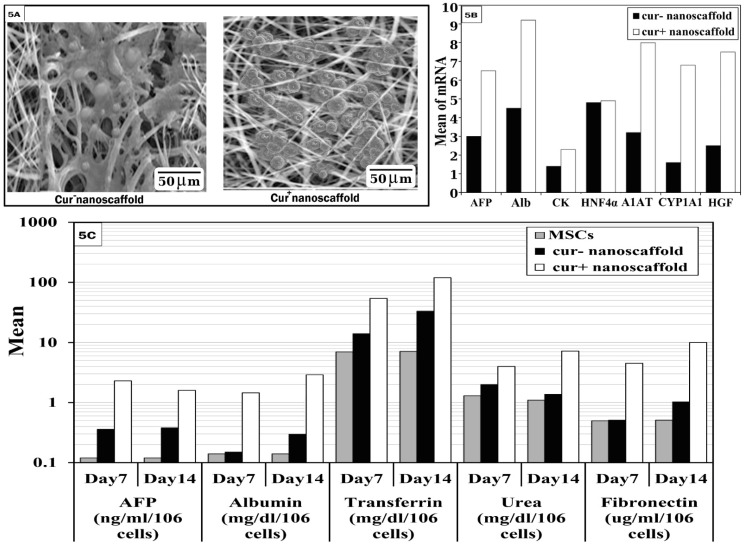
*In-vitro* differentiation of MSCs on both scaffolds.

SEM examination of both scaffolds showed change of fibroblastoid cells into ovoid and polygonal cells, additionally cells on cur^+^nanoscaffold showed more attachment and appeared in aggregate with strong cell-cell contact and tight junction on the scaffold (Figure 5A).

On molecular level, the results showed significant higher gene expression of AFP, ALB, CK18, HNF4α, alfa-1 antitrypsin (A1AT), CYP1A1 and HGF in cur^+^ nanoscaffold group than cur^-^ nanoscaffold. Moreover, non-induced cells showed no expression of hepatic specific genes (Figure 5B).

We assessed the functional profile of hMSCs derived hepatocytes, as shown in Figure 5C, differentiated cells on cur^+^ nanoscaffold significant higher level of AFP on both day 7 and day 14 than cur^-^ nanoscaffold (p < 0.001), also AFP increased on day 7 and then decreased on day 14 in cur^+^ nanoscaffold in comparison to cur^-^ nanoscaffold which continue to be high until day 14. Albumin, transferrin, fibronectin and urea synthesis were significantly increased on day 14 than day 7 in both nanoscaffolds, there was no significant difference between control cells (undifferentiated MSCs) and cur^-^ nanoscaffold regarding albumin and fibronectin measured on day 7 which are increased significantly on day 14 in cur^-^ nanoscaffold and there was significant higher level regarding transferrin and urea in cur^-^ nanoscaffold than control in both days7 and 14. Differentiated cells on cur^+^ nanoscaffold present significant higher level of Albumin, transferrin, fibronectin and urea on all times when compared with both control and cur^-^ nanoscaffold (p < 0.001).

### Immunohistochemical analysis and hepatic gene expression in liver tissue after cell transplanation

**Figure 6:**
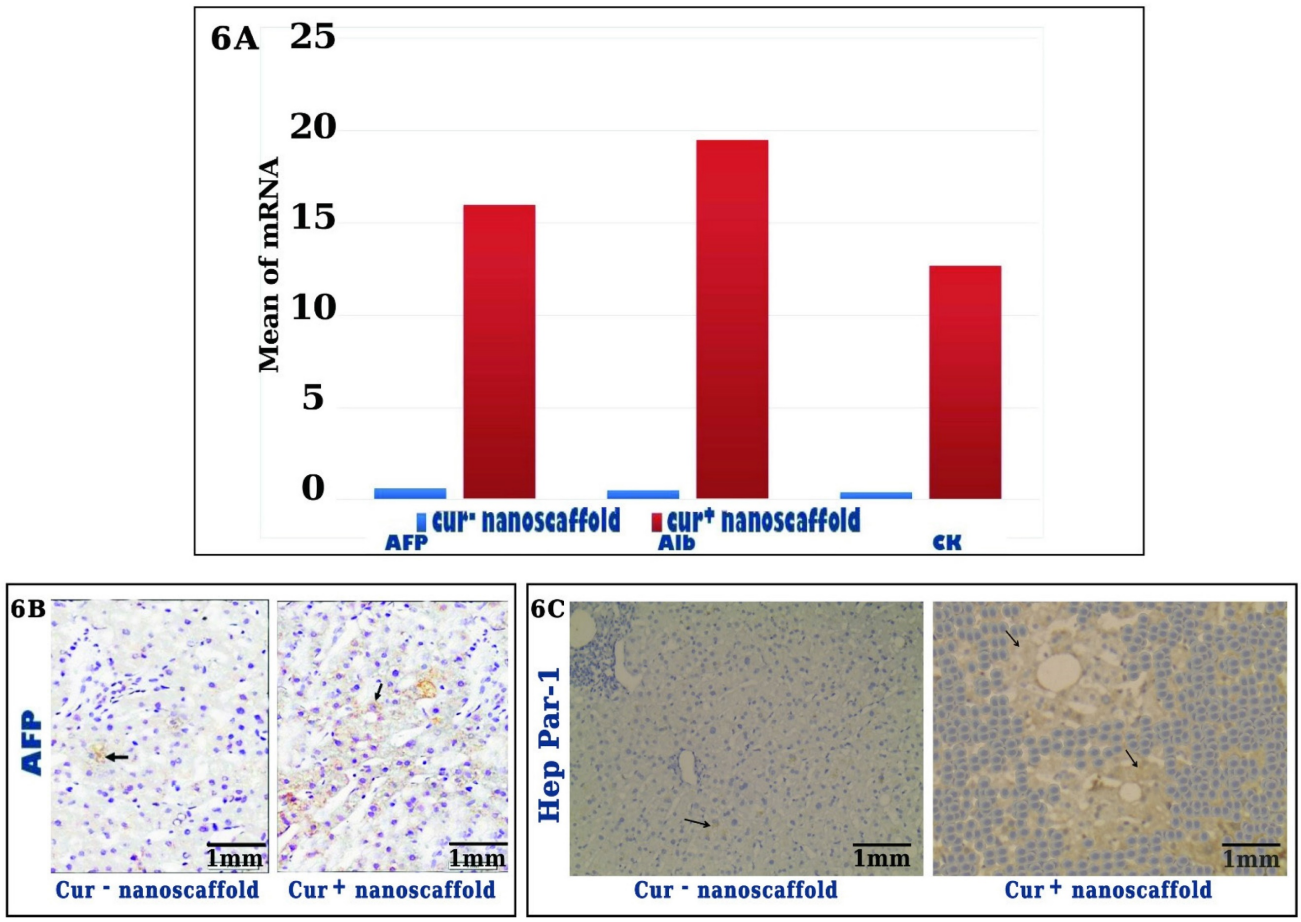
Assessment of the fate of injected cells by gene expression (fig.6A) and immunohistochemical analysis, black arrow showed the differentiated cells (fig.6B).

The fate of injected hBM-MSCs from both nanoscaffold cultures and its ability to differentiate into functional hepatocytes were evaluated by immunohistochemistry analysis and qRT-PCR. The staining of human -specific AFP is brownish, granular and cytoplasmic, the expression of human -specific Hep Par-1 is diffuse cytoplasmic staining. In cur^-^ nanoscaffold treated group liver showed scattered positive reaction for the human -specific AFP and Hep Par-1 which accounts very small percentage of the section about 2-3% of section, group treated with cur^+^ nanoscaffold showed diffuse positive reaction mainly around the central vein accounting about 18-26s% of section (Figure 6A&B). The mRNA level of human specific genes AFP, ALB and CK18 were significantly higher in cur^+^ nanoscaffold than cur^-^ nanoscaffold (p < 0.001) (Figure 6C).

#### cur^+^ nanoscaffold/MSCs downregulate the fibrogenic genes in liver tissue

**Figure 7:**
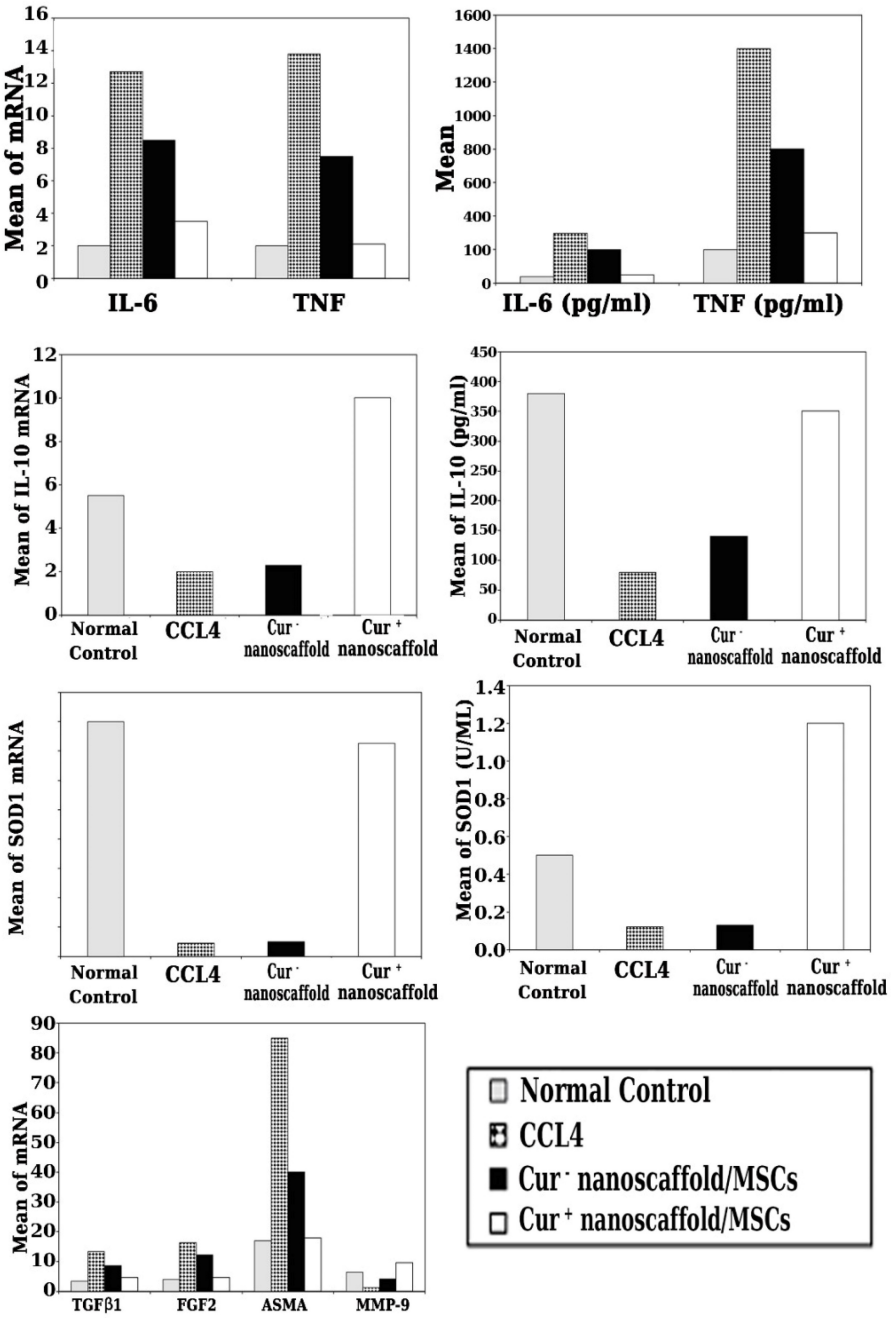
Impact of cur^+^ nanoscaffold/MSCs on inflammatory, oxidative stress status and fibrogenic genes of fibrotic liver.

To assess the molecular mechanism of reduction of fibrosis by cur^+^ nanoscaffold/MSCs, we analysed gene expression of TGFβ1, FGF-2, α-SMA and MMP-9 and we found that significant upregulation of these fibrogenic genes with down regulation of MMP-9 gene in control group, after cur^+^/nanoscaffold /MSCs treatment there was with dramatic reduction of fibrogenic genes and marked up regulation of MMP-9 than cur^-^ nanoscaffold/MSCs (p < 0.001) Figure 7.

### Impact of cur^+^ nanoscaffold/MSCs on inflammatory and oxidative stress status of fibrotic liver

To explore the effect of transplanted preconditioned cells by curcumin nanoscaffold on the inflammatory milieu of fibrotic liver, mRNA level of inflammatory and anti-inflammatory cytokines were quantified using qRT-PCR and the results showed that significant up regulation of mRNA of TNF-α, IL-6 and down regulation of IL-10 in CCl4 group, in cur^+^ nanoscaffold/MSCs treated group showed marked reduction in mRNA of TNF-α, IL-6 and significantly upregulate of IL-10 genes than the control and cur^-^ nanoscaffold/MSCs (p < 0.001) (Figure 7). We also measured the circulating cytokines in the experimental groups and there was significant lower level of TNF-α & IL-6 and higher level of IL-10 in cur^+^ nanoscaffold/MSCs than the control and cur^-^ nanoscaffold/MSCs treated groups (p < 0.001) Figure 7.

**To elucidate if** transplanted preconditioned cells by curcumin nanoscaffold have effective role in oxidative stress, expression of antioxidant SOD1gene and measurement of circulating SOD1 antioxidant enzyme were evaluated. As shown in Figure 7, cur^+^ nanoscaffold/MSCs have the ability to significantly increase the expression of SOD1 gene and increased the circulating level of SOD1 activity compared to cur^-^ nanoscaffold/MSCs treated groups which appeared as it has no role in modulation the oxidative stress condition.

## Discussion

Due to the certain properties of cur, preparation of ideal nanfibrous scaffold for release of cur in a controlled manner still questionable. Nanfibrous scaffold as delivery system compared with other cur carriers has the advantages of easy technique, inexpensive, better loading ability, multiple compounds of polymer to optimize the drug bioactivity and cellular function can be used and control cur release can be achieved 19.

In the present study, cur^+^/ collagen/PCL/PLGA and cur^-^/ collagen/PCL/PLGA were prepared and characterized to assess their properties which affect the cur release that will reflected on the MSCs function. We used natural and synthetic polymers with dual electrospinning to optimize the scaffold. The cur characterized by hydrophobicity and low bioavailability, so we measured the hydrophilicity which is important in both cell adhesion and bioavailability of cur and our results showed that cur^+^/nanoscaffold has lower hydration than cur^-^/nanoscaffold in first 12 hrs which may due to the hydrophobic property of cur but after 24hrs, no difference between the both scaffolds and this indicate that the usage of collagen improve the water absorption of cur^+^/nanoscaffold with more culture time. In previous study by Golchin et al. 17 showed that use of cur^+^/CS/PVA‐carbopol/PCL scaffold increase the water absorption of cur but still lower than cur ^-^ CS/PVA‐carbopol/PCL scaffold.

The zeta potential which reflects the negative charges of the nanofiber has a critical role in solution stability of the nanofiber and the previous studies showed that instability with zeta above ±60 mV and particle aggregation with zeta below ±5 mV and values in between have enough stability 20, in our study cur^+^/nanoscaffold showed more negative zeta potential than cur^-^/nanoscaffold and that indicate that the cur increase repulsive charge between the fibres. It is reported that negative zeta potential has positive impact on cell proliferation and attachment 21. Additionally, the cur release kinetics showed low concentration in the first 24hrs then increased in time dependent manner until the 4^th^ day then become constant until the end of the culture period, this kinetic may be explained by the hydrophobicity of fibre in first 24hrs and the release increase with increased the hydrophilicity, our results are supported by previous studies which reported increase of cur release with time [17&22].

Recent studies used MSCs therapy in improving liver fibrosis but the magnitude of the results is inconsistent and may be due to decreasing the survival and engraftment in the inflammatory environment in liver fibrosis. So, if a small percentage remain in the injured tissue and can produce some effects, it is suggested that if these small percentage of cells are able to produce such effects, then increasing the migration, survival, engraftment and biological function of the cells by preconditioning is critical for their clinical effectiveness. Different formulations of Cur for preconditioning of MSCs was used in some studies to improve the therapeutic efficiency as in wound healing 22, bone regeneration 15, osteoarthritis 23 but according to our knowledge, no study investigated the cur preconditioning of MSCs to treat liver fibrosis.

Use of stem cell therapy in liver fibrosis still has certain challenges, such as time of response especially in patients with critical liver failure, the efficacy and how to maintain of this efficacy to prevent failure of remission. The results in the current work showed that usage of cur/collagen/PCL/PLGA scaffold has faster, ameliorative and protective effect than the wild MSCs in animal model of liver fibrosis.

We assessed the improvement of fibrosis at 1^st^ week, 4^th^ week, 12^th^ week and 24^th^ week of cell infusion, we observed faster and ameliorative effect in cur^+^/nanoscaffold treated group than cur^-^/nanoscaffold treated rats as in cur^-^/nanoscaffold there was no improvement in fibrosis level at 1 week of cell infusion compared to reduction in fibrosis from 15% to 3.6% in cur^+^/nanoscaffold which ameliorated on 4^th^ week evidenced by disappearance of septal collagen deposition by histopathological examination and dramatic reduction in hydroxyprolin content compared to reduction to 6.5% at the 4^th^ week in cur^-^/nanoscaffold group. Also, this effect was confirmed by molecular level by quantitation of collagen and ASMA mRNA which showed reduction by 6.0 fold at 1 week and 19.0 fold at 4^th^ week compared to 1.0 and 1.8 fold in cur^-^/nanoscaffold group and this faster and ameliorative effect in animals receiving primed MSCs was also reflected on restoration of liver function.

In the current study, we like to assess if curcumin preconditioning of MSCs can maintain this ameliorative effect and protect against return of the fibrosis which actually represent a critical challenge in use MSCs in therapy and still there is a controversy about the long term efficacy of stem cells, there many preclinical and clinical studies prove the reduction of liver fibrosis by MSCs injection 24-26 but maintaining its effect were achieved after many times of cell injection up to 9 injections in one experiment 27 and partially achieved in another studies 28-29 and not achieved at all in others 30-31. So, we investigated the fibrosis degree on 12^th^ week and 24^th^ week of cell infusion and we found that no significant difference compared to that at 4^th^ week in cur^+^/nanoscaffold and this indicates that curcumin preconditioning of MSCs maintained the ameliorative effect of the primed MSCs and protected against the return of fibrosis compared to cur^-^/nanoscaffold which showed a return of fibrosis from 6.5% to 10.4% . The increase in hydroyproline content and deterioration of liver function again this indicate that wild MSCs have not the ability to maintain their therapeutic effects.

Previous studies reported that the reduction of hepatic fibrosis by MSCs are achieved by multiple mechanisms including release of trophic factors by transplanted cells, down regulation of anti-inflammatory cytokines, hepatocyte apoptosis and by differentiation of transplanted cells into functioning hepatocytes 32. In the current study, cur preconditioning enhanced proliferation, migration, differentiation and the survival of MSCs. The proliferative capacity of cur- treated cells increased gradually over time to reach the maximum difference on day 8 of culture by 7.9 fold than wild cells and this due to gradual release of cur, which become constant during the remaining period of culture. In agreement with our result, Yousefi et al reported that cur at lower concentration (0.5-5.0 μM) upregulated the proliferation of MSCs and at higher concentration > 12 μM decreases the MSCs growth 33. The very small amount of the injected cells that reach the injured liver was a challenge of using MSCs therapy, in the present study cur treated cells showed much more migration capacity than which tested* in-vitro* by scratching wound and in -vivo by quantitation of male SRY gene in the female rats to reach 22.4 fold than the wild MSCs and this indicates that cur preconditioning enhanced the homing and engraftment of MSCs in the required site. Recent study by Azam et al. reported that cur priming of MSCs enhanced its migration in treatment of burn wound 34.

There is controversy about the ability of the transplanted MSCs to differentiate into functioning hepatocytes, some studies reported its ability to differentiate into hepatocytes 35. Another study proved no differentiation of the injected cells 36 and other reports stated that trans-differentiation of MSCs to hepatocytes rarely happened (less than 1%) in relation to the amount injected 39. Previous reports indicated that cur induce both osteoblastic and chondrogenic differentiation but inhibit adipogenic differentiation of MSCs 40. In the current study, liver tissues of rats treated with cur^+^/nanoscaffold primed MSCs showed much higher expression of human specific genes of AFP, ALB and CK18 by 35.6 fold than rats treated with cur^-^/nanoscaffold and this also confirmed by immunohistochemistry and this one of the explanation the effective therapy of cur treated cells. This in-vivo effect was confirmed by the *in-vitro* experiment, induced cur^+^/nanoscaffold primed MSCs showed significantly higher albumin, AFP, transferrin, fibronectin and urea levels in culture supernatant than cur^-^/nanoscaffold culture and on molecular level, cur^+^/nanoscaffold primed MSCs have significant higher mRNA level of hepatocyte specific genes than cur^-^/nanoscaffold MSCs culture.

The senescence of MSCs is an obstacle against its clinical application, in the current study the survival of cur^+^/nanoscaffold primed MSCs was observed up to 24^th^ weeks in the liver tissue mainly around the portal vein compared to 12^th^ week in rats treated with cur^-^/nanoscaffold MSCs (Khuu et al reported that only small portion of MSCs engraft the liver after surgical hepatectomy and persist only for 8 weeks 41 and this may be due to the high efficacy of cur to enhance the migration and proliferation of the cells and this could explain the maintaining and protective efficacy of cur^+^/nanoscaffold primed MSCs. Supportive to our results, a recent study reported that cur ameliorate the senescence of MSCs through modulating autophagy pathway 42.

It is observed that the paracrine release ability of cur^+^/nanoscaffold primed MSCs was increased compared to cur^-^/nanoscaffold MSCs and this increase contributed to faster and effective regeneration because HGF and EGF increase the hepatocyte proliferation and differentiation, VEGF responsible for angiogenesis and then liver regeneration in agreement with previous study [5&34].

Previous studies demonstrated that the underlying molecular mechanism of reduction of fibrosis by MSCs through inhibition of collagen deposition and down regulation of transforming growth factor- β1 (TGFβ1), the main isoform of TGF-β, is included in the process of fibrosis in many organs, including the liver through activation of fibrogenic genes with proliferation hepatic stellate cells (HSCs) and over expression of matrix metalloproteinase (MMP) especially MMP-9 which directly degrades the extracellular matrix and induces hepatic stellate cells 43. So, we analysed TGFβ1, FGF-2, collagen type I, ASMA and MMP-9 and we found that upregulation of these fibrogenic genes with down regulation of MMP-9 gene in control group. Interestingly, in cur^+^/nanoscaffold /MSCs treated group, there was with dramatic reduction of fibrogenic genes and marked up- regulation of MMP-9 than cur^-^/nanoscaffold /MSCs and this finding was supported by histological aspect of liver tissue when examined by H&E and MT, which revealed that the much superior ability of cur^+^/nanoscaffold /MSCs to reduce collagen deposition.

Prolonged injury of the liver leads to hepatic parenchymal cell death, damage-associated molecular patterns (DAMPs), released from the dying cells initiate inflammatory response which further activate the hepatic stellate cells as tumor necrosis factor (TNF), interleukin 1 beta (IL-1b), interleukin-6 (IL-6) with further recruitment of inflammatory cells and more damage of hepatocytes 43. Our results are also in agreement as we noticed that in control group showed high level of circulating IL-6 and TNF and low level of anti-inflammatory IL-10 and this confirmed on the molecular level from liver tissue. Interestingly, after cell infusion of cur^+^/nanoscaffold/MSCs treated rats showed significant depletion of inflammatory cytokines and up regulate anti-inflammatory ones in comparison with cur^-^/nanoscaffold /MSCs. It is of note that MSCs have intrinsic capacity to decrease inflammation as observed in many studies 44, however the observed marked decrease in inflammatory phase may be due to enhanced migration, proliferation and survival of cur^+^/nanoscaffold primed MSCs in the liver.

Persistent of inflammatory cells produce reactive radicals which cause oxidative imbalance which negatively affects the regeneration process 45. Super oxide dismutase (Sod1) is anti-oxidant enzyme which protects the cells from oxidative damage 45. There was no any difference between the control group and group treated with cur^-^/nanoscaffold /MSCs. Conversely, cur^+^/nanoscaffold /MSCs treated group showed higher expression of Sod1which indicates that cur preconditioning of MSCs neutralizes the oxidative radicals by upregulation of antioxidant enzymes. This result is in agreement with previous studies which reported that cur preconditioning of MSCs enhances the antioxidant ability of MSCs 34;46.

## Conclusion

Inconclusion, we developed curcumin/collagen/PCL/PLGA nanoscaffold with control release of cur to elicit the MSCs to ameliorate CCL-4 induced liver fibrosis. Our results demonstrated that cur+/ nanoscaffold preconditioning of MSCs contribute to the efficacy of injected MSCs to survived for longer time and to make its paracrine effect for longer time with enhancement of their hepatogenic differentiation resulted in faster, ameliorative and protective capacity against liver fibrosis. So, the outcomes of this study enhance the potential clinical utility of cur^+^/ nanoscaffold preconditioning of MSCs for treatment of liver fibrosis. It is recommended for further studies to investigate how curcumin acted on subcellular level for more understanding its therapeutic effect.

## Figures and Tables

**Table 1:** Primers of PCR

Primer	Sequence	Annealing temperature
Collagen-1	F: 5'-AATTGGAGCTGTTGGTAACGC-3 R: 5'-CACCAGTAAGGCCGTTTGC-3	54°C
SRY	F: 5'-GCG ACC CAT GAACGCAT-3 R:5'- AGT TTC GCA TTCTGGGATTCT CT-3 Probe: FAM-TGG TCT CGC GAT CAGAGG CGC-TAMRA	59°C
β-actin	F: 5'-TCACCCACACTG TGCCCATCTACG A-3 R: 5'-CAGCGG AAC CGCTCATTG CCA ATG G-3 Probe: FAM-ATGCCCTCCCCCATGCCATCCTGCGT-TAMRA.	59°C
AFP	F: 5'-GATGCACCTGACCCACTTTATAAA-3 R: 5'-GAGATTGTCTGACCGATTCACT-3	58°C
ALB	F: 5'-CAACTATGTCCGTGAGCTTCCA-3 R: 5'-GTGGTCGGTGCTGGTCTATATG-3	58°C
CK-18	F: 5'-CCC GCT ACG CCC TAC AGAT-3 R: 5'-ACCACTTTGCCATCCACTATCC-3	58°C
HNF4α	F: 5'-ACTACATCAACGACCGCCAGT-3 R: 5'-ATCTGCTCGATCATCTGCCAG-3	58°C
A1AT	F: 5'-AGGTGCCTATGATGAAGCGT-3 R: 5'-TGGCAGACCTTCTGTCTTCATT-3	58°C
CYP1A1	F: 5'-CAAGGGGCGTTGTGTCTTTG-3 R: 5'-GTCGATAGCACCATCAGGGG -3	58°C
HGF	F: 5'-ATGAGAGAGGCGAGGAGAAAC-3 R:5'-GTAGCCCCAGCCGTAAATACT-3	58°C
TGFβ1	F: 5'-GCCCTGGATACCAACTATTGC-3 R: 5'-TGTTGGACAGCTGCTCCACCT-3	58°C
FGF	F: 5'-TGACGGGGTCCGGGAGAAGA-3 R: 5'-ATAGCCAGGTAACGGTTAGCACACAC-3	58°C
*MMP-9*	F: 5'-AGCGAGGTGGACCGGATGTT-3 R: AGAAGCGGTCCTGGCAGAAATAG-3	58°C
αSMA	F: 5_- GTGCTATGTCGCTCTGGACTTTGA R: 5_- ATGAAAGATGGCTGGAAGAGGGTC-3	58°C
IL-6	F: 5'-CCACCCACAGACCAGTA-3 R:5'-CTCCAGAAGACCAGAGCAGAT-3	58°C
TNF-α	F: 5'-TGCCTCAGCCTCTTCTCATT-3 R:5'-GCTTGTGGTTTGCTACGAC-3	58°C
IL-10	F: 5'- CGAAGCTTGCCATGCTTGGCTCAGCAC-3′ R: CGTCTAGATCAATTTTTCATTTTGAGTG-3′	61°C
SOD1	F: 5'TGCTTTTTGCTCTCCCAGGT-3 R:5'CTGGACCGCCATGTTTCTTA-3	58°C
GADPH	F: 5'-GCATGGCCTTCCGTGTTC-3 R: 5'-GATGTCATCATACTTGGCAGGTTT-3	58°C

## Abbreviations

MSCs: Mesenchymal Stem Cells
cur: curcumin
HGF: hepatocyte growth factor
EGF: epidermal growth factor
VEGF: Vascular endothelial growth factor
ALT: Alanine transferase
AST: Aspartate transferase
SRY: sex-determining region Y
PCNA: proliferating cell nuclear antigen
SOD: superoxide dismutase
PLGA: poly lactic-co-glycolic acid
PCL: polycaprolactone
